# Clinical Performance of Extra-Short (≤5.5 mm) Compared to Longer Implants Splinted under the Same Prosthesis: A Randomized Clinical Trial

**DOI:** 10.3390/dj12090292

**Published:** 2024-09-13

**Authors:** Eduardo Anitua, Adriana Montalvillo, Asier Eguia, Mohammad Hamdan Alkhraisat

**Affiliations:** 1 University Institute for Regenerative Medicine and Oral Implantology—UIRMI (UPV/EHU-Fundación Eduardo Anitua), 01007 Vitoria, Spain; aeguiadelvalle@gmail.com (A.E.); dr.khraisat@gmail.com (M.H.A.); 2 BTI Biotechnology Institute, 01005 Vitoria, Spain; 3 Department of Cellular Biology and Histology, Faculty of Medicine and Nursing, Universidad del País Vasco/Euskal Herriko Unibertsitaea (UPV/EHU), 48940 Leioa, Spain; amontalvillo88@gmail.com; 4 Eduardo Anitua Dental Clinic, 01007 Vitoria, Spain; 5 Oral and Maxillofacial Surgery, Oral Medicine and Periodontics Department, Faculty of Dentistry, University of Jordan, Amman 11942, Jordan

**Keywords:** dental implant, short implants, RCT, marginal bone loss, implant survival

## Abstract

**Objective:** This randomized controlled split-mouth trial compared the performance of 5.5 mm length implants (test group; TG) splinted within the same fixed prosthesis as longer implants (≥6.5 mm; control group; CG) in posterior regions. **Methods:** The primary hypothesis was that implant length does not affect marginal bone loss (MBL) one year post-implantation, while the secondary hypotheses included implant survival, peri-implant clinical variables, and prosthetic complications. Fifteen patients (eight males, seven females) with a mean age of 67 ± 9 years were included. **Results:** No significant difference in the implant position between groups (*p* = 0.808) was observed. Implant diameters ranged from 3.00 to 4.25 mm, and the most common bone type was type I (67%). Bone density (*p* = 0.574) and implant insertion torque (*p* = 0.888) were similar between groups. Mesial MBL (mean: −0.1; range: −1.19 to 0.24 for TG, and −0.03; −1.75 to 0.45 for CG; *p* = 0.955) and distal MBL (mean: −0.05; range: −1.41 to 0.27 for TG, and 0.08; −1.45 to 0.72 for CG; *p* = 0.118) did not show statistical differences. There were no implant failures or technical complications. **Conclusions:** These findings suggest that 5.5 mm length implants could be a viable option for use in posterior regions, providing similar clinical outcomes to longer implants one year post-implantation.

## 1. Introduction

Tooth extraction results in bone resorption and subsequent dimensional changes in the alveolar ridge [1]. In the absence of any procedure [2], edentulism leads to different degrees of both vertical and horizontal atrophy due to a lack of masticatory function [3]. This bone resorption often hinders prosthetic rehabilitation with dental implants when there is insufficient residual bone volume for implant placement [4]. Ancillary surgeries for bone augmentation are frequently required [5]. Focusing on vertical atrophy, common techniques include sinus lift [6], vertical bone augmentation in the mandible [7], and inferior alveolar nerve repositioning [8], generally yielding satisfactory outcomes. However, these techniques have disadvantages: they significantly increase treatment costs and duration [9], they are associated with higher patient morbidity compared to simpler implant surgeries [9], and they carry greater risks of post-surgical complications such as sinus membrane perforation, graft exposure and failure, and sinusitis [10], or even neurosensory complications from inferior alveolar nerve repositioning [8].

The placement of shorter dental implants has emerged as a viable alternative to traditional bone augmentation surgeries in patients with vertical bone atrophy, as they contribute to a reduction in surgical complexity, lower morbidity, and shorter treatment duration [9,11]. Clinical studies have demonstrated that, despite their reduced length and increased crown/implant ratio, they can achieve similar or better success rates, survival rates, and peri-implant bone stability than longer implants placed with or without bone grafting procedures [4,6,9,12,13,14]. Furthermore, short implants have demonstrated high predictability in the long term, even after 10 years of follow-up [15,16]. A systematic review and meta-analysis of RCTs [4] found that short implants (≤7 mm) in the deficient posterior mandible had comparable outcomes in terms of implant survival, MBL, and complications to regular implants (>7 mm) placed after vertical bone augmentation within a 5-year period. Conversely, an RCT comparing the performance of short (6 mm) versus long (10 mm) dental implants in the posterior areas over 5 years showed a higher survival rate for the long implants group. While the crown/implant ratio was significantly higher in the short group, there was no significant difference in MBL between both groups. Additionally, neither the higher C/I ratio nor the implant length significantly affected complication rates [17].

The classification criteria for short implants have continually evolved. Different authors defined short implants variably; some categorize them as implants shorter than 10 mm [18], while others used a threshold of 8 mm [19]. However, more recent classifications have set the limit at 6.5 mm [20] or even 6 mm [14].

A recent systematic review (SR) [6] including randomized controlled trials (RCTs) compared the performance of short (≤6 mm) and long (≥10 mm) dental implants and assessed implant survival, marginal bone loss, and complications in different clinical scenarios. This study showed similar 5-year survival rates for short and longer implants in non-augmented bone and full-mouth rehabilitation in both jaws, as well as for short implants in the maxilla as an alternative to sinus lift. Both marginal bone levels and complications were comparable. The clinical performance (with 5 years of follow-up) of ≤6 mm short implants, supporting single crowns or short fixed partial dentures, has also been assessed by another recent SR [21]. Good durability and low and minimal-complexity (mostly screw-loosening) complication rates were observed.

Another SR [22] compared the effectiveness of extra-short (<6 mm) implants to standard implants (≥8 mm) associated with bone grafting and revealed that the risks of implant loss and prosthetic complications were similar between both groups. However, biological complications were significantly lower for extra-short implants, which also demonstrated greater peri-implant bone stability at the 12-month follow-up. The same risk of failure for single crowns supported by extra-short implants (≤6 mm) or those supported by longer implants, regardless of previous maxillary sinus augmentation or follow-up period, was observed in the SR performed by Badaró et al. [23].

When comparing extra-short implants (≤6 mm) to lateral sinus floor augmentation with regular implants (≥10 mm) in the posterior maxilla, a SR and meta-analysis by Grunau et al. [24] found no significant differences in implant loss rates at 1 and 3 years. At 5 years, regular implants had a nearly significant lower risk of implant loss. However, short implants exhibited significantly lower mean marginal bone loss at all follow-up periods, demonstrating comparable outcomes to regular implants in the atrophic posterior maxilla within the first 5 years.

A 10-year prospective case-series study [25] explored the long-term effectiveness of 6 mm short implants for single restorations in the resorbed posterior mandible. The study reports a 100% implant survival rate, minimal marginal bone loss (0.18 mm; SE: 0.08), and consistently low clinical indices for peri-implant tissue health (plaque, calculus, gingiva, and bleeding indices). Additionally, patient satisfaction remained high throughout the study. 

Although there is available evidence in the literature supporting the favorable clinical performance of extra-short implants, different aspects require further in-depth clarification. This randomized clinical study aimed to further study the clinical behavior of 5.5 mm length bone-level implants by prospectively comparing them with longer implants, splinted under the same FPDs within the same patients. The primary hypothesis was that implant length does not affect marginal bone loss. The secondary hypotheses were (1) that implant length does not affect implant survival or peri-implant clinical variables such as Bleeding on Probing (BoP) and Probing Depth (PD) and (2) that implant length does not affect the occurrence of prosthetic complications.

## 2. Materials and Methods

### 2.1. Trial Design

This clinical research involved a randomized controlled split-mouth trial comparing the performance of 5.5 mm length implants (test group; TG) to ≥6.5 mm length implants (control group; CG) in posterior regions. Both the test and control implants were splinted within the same fixed prostheses (FPDs). The randomization determined the specific position for each implant and a prospective observational study design was employed. This approach allowed for paired analysis, comparing intra-subject performance, and contributed to a reduction in confounding factors related to biological variables. To reduce inter-observer variability, bone stability measurements from radiographic records were taken by a single, experienced investigator using consistent criteria. 

The trial was registered at clinicaltrials.gov under the ID NCT04929743.

### 2.2. Ethical Considerations

This study was conducted in accordance with the ethical principles outlined in the Declaration of Helsinki and received approval from the Ethics Committee of Research with Medicaments (CEIm) of the Basque Country (PS2020061). All participants provided written informed consent prior to their inclusion in the study, ensuring their voluntary participation and understanding of the research procedures, potential risks, and benefits involved. The confidentiality and anonymity of the participants were maintained throughout the study.

### 2.3. Participants

The study population consisted of patients with partial edentulism. The following criteria were considered to include or exclude potential participants:

#### 2.3.1. Inclusion Criteria

- Age ≥ 18 years.
- Clinical indication for placing a FPD supported by a maximum of 4 implants in the posterior regions.
- Sufficient bone height to place implants of at least 6.5 mm in length.
- Availability for follow-up observation.
- Signed informed consent.

#### 2.3.2. Exclusion Criteria

- Need for bone-augmentation surgery prior to implant placement.
- Smoking > 10 cigarettes/day.
- Patients with poorly controlled diabetes.
- Patients on chronic treatment with non-steroidal anti-inflammatory drugs (NSAIDs).
- Patients on oral or intravenous bisphosphonates.
- Patients undergoing chemotherapy or radiotherapy.
- Patients on systemic corticosteroids.

### 2.4. Randomization

The sealed envelope system [26] was employed to ensure random allocation of dental sites to the two study groups in this randomized controlled split-mouth trial. The following measures were taken to minimize selection bias and ensure that the randomization process was robust, thereby enhancing the reliability and validity of the study outcomes.

#### 2.4.1. Sequence Generation

The randomization sequence was generated using a computer-based random number generator to ensure equal allocation of dental sites to the two study groups. The randomization aimed to compare the performance of 5.5 mm length implants (TG) to ≥6.5 mm length implants (CG) within a split-mouth design. Each patient’s implant-requiring sites were divided into two halves, with one half randomly assigned to receive the test implant(s) and the other half to receive the control implant(s).

#### 2.4.2. Allocation Concealment Mechanisms

To ensure allocation concealment, the sequence generated was placed into opaque, sealed envelopes. These envelopes were numbered sequentially and kept securely until the moment of the surgical procedure. The envelopes were prepared and sealed by a staff member who was not involved in the clinical assessments or implant placements to maintain blinding of the allocation process.

#### 2.4.3. Implementation

The allocation sequence was implemented by the principal investigator. At the time of surgery, the principal investigator opened the next envelope in the sequence to determine the assignment of the implant type to each site of the patient’s mouth. This process was repeated for each patient to maintain randomization and ensure unbiased allocation. The surgical team, who performed the implant placement, adhered strictly to the allocation revealed by the envelopes.

### 2.5. Interventions

All the patients received local anesthesia and a full-thickness flap was raised to allow the implant placement. All the implants used in this study presented the same surface treatment (UnicCa^®^ surface, BTI Biotechnology Institute, Vitoria, Spain). The implant length in each position (5.5 or ≥6.5) was determined by randomization. A low-speed drilling protocol [27] was employed to prepare the implant bed for all the implants in both the CG and TG. After the implant placement, the insertion torque and bone type at the insertion site were recorded. When the insertion torque was ≥25 N.cm and the bone type was I, II, or III, implants were immediately loaded. In all these cases, a screw-retained on intermediate abutment, metal-resin veneered, provisional FPD was used.

When the torque and bone type criteria were unsuitable for immediate loading, a delayed loading procedure with a definitive metal–ceramic, screw-retained FPD restoration was employed after submerged osseointegration. A second surgery after 12 ± 2 weeks was needed to open a full-thickness flap to remove the implant cover in such patients. For both immediate loading and delayed loading, the same design of intermediate abutments was employed (Multi-IM^®^ transepithelial abutments; BTI Biotechnology Institute, Vitoria, Spain).

During the follow-up visit one year after implantation, clinical measurements (PD and BoP) were performed with the aid of a periodontal probe. PD was measured from the gingival margin to the bottom of the peri-implant sulcus, recorded at six positions around each implant (mesiobuccal, midbuccal, distobuccal, mesiolingual, midlingual, and distolingual). Periapical radiographs were obtained at the implant placement visit, the loading visit, and the one-year follow-up visit.

#### The Following Visit Schedule Was Employed for All the Participants

First Visit (V1, time ≤ 0): Patients eligible under the inclusion/exclusion criteria were invited to join the study. After participation agreement, further aspects of the investigation were explained, an informed consent was obtained, and a participant code was assigned. Demographic and other relevant data were recorded in the Case Report Form (CRF) and if no previous appropriate radiographic study was available, an oral CBCT was obtained for a proper diagnosis and planification of the treatment.

Implant Placement Visit (V2, time = 0): Before the surgery, the randomization process determined the required implant lengths for each position (5.5 mm length implant or ≥6.5 mm length implant). The insertion torque, the bone density, and the type of bone at the implant placement sites were recorded, along with the positions and dimensions of the test and control implants (platform, length, and width). All the implants placed in the study were restored using screw-retained prostheses with an immediate or delayed (after 12 weeks) loading protocol. Immediate loading was performed in those implants with an insertion torque of ≥25 N.cm and placed in bone types I, II, or III. A periapical radiograph of the implants was obtained post-surgery, and any technical or biological complications were recorded.

Delayed Loading Visit (V3, 12 weeks ± 2 weeks post-implantation): Implants unsuitable for immediate loading were restored during this visit. Complications and medication changes were recorded, and a post-loading periapical radiograph was taken.

Follow-up Visit (V4, time = 1 year ± 2 months post-implantation): One year post-implant insertion, the patient attended a follow-up visit where a periapical radiograph and clinical measurements (PD and BoP) were obtained. Any complications or medication changes during the study were also recorded.

### 2.6. Outcomes

The main objective of this clinical investigation was to determine the predictability of 5.5 mm length implants compared to 6.5 mm length implants splinted in the same prosthesis. For this reason, the primary study outcome was the marginal bone loss one year post-implantation and secondary study variables were implant survival one year post-implantation, clinical evolution variables (BoP or PD), and occurrence of prosthetic/implant complications.

### 2.7. Sample Size Calculation

A previous study [28] evaluating 4 mm length implants compared to longer implants observed a difference between groups of 0.16 mm in bone loss with a standard deviation of 0.161. Considering this difference as the true difference, a paired analysis with a test ratio of 1:1 requires 13 pairs of subjects to accept the null hypothesis that there are no significant differences between groups with a type 1 error of 0.05 and a statistical power of 0.9. Accounting for a 20% follow-up loss, the calculated sample size was 15 subjects.

### 2.8. Blinding

In the present study, blinding was not feasible due to the nature of the surgical procedure involved. During the placement of dental implants, the surgeon had to be aware of the specific type of implant being used to ensure proper handling, drilling, and placement. Consequently, the surgeon could directly observe and identify the implant type during the procedure, making blinding impractical. To mitigate potential bias, the study incorporated several measures. First, the surgical team was trained to adhere strictly to standardized protocols for implant placement, ensuring uniformity across all cases. Second, outcome assessments were conducted by an independent examiner who was not involved in the surgical procedures. Third, data analysis was performed by a statistician who was also blinded to the treatment groups. These steps were implemented to minimize bias and enhance the reliability and validity of the study results despite the inability to blind the surgical team and the examiner who conducted the clinical assessment.

### 2.9. Statistical Methods

An intention to treat analysis was performed. Categorical variables were described by calculating the frequency and were compared between the study groups with Chi square and Fisher’s exact tests. The normality of the data distribution for the quantitative data was tested by the Shapiro–Wilk test. For normally distributed variables, the mean and standard deviation were calculated and the comparison between the study groups was performed with a paired Student test. Otherwise, the median and the range were calculated and the comparison between the study groups was performed by the Wilcoxon test. The statistical analysis was performed in IBM SPSS Statistics (Armonk, NY, USA). The threshold of statistical significance was at *p*-value < 0.05.

### 2.10. Report/Publication Guidelines

To accurately report the study results, the CONSORT 2010 statement’s updated guidelines for reporting parallel group randomized trials were employed [29].

## 3. Results

**Figure 1:**
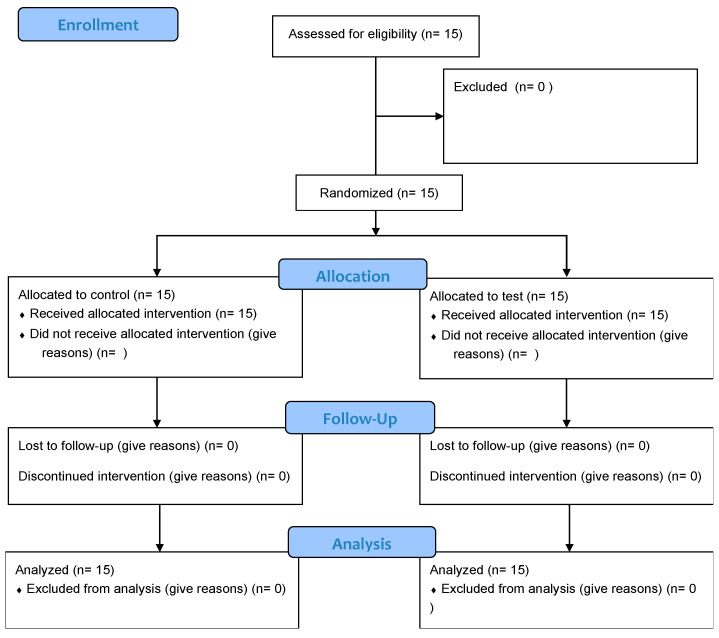
CONSORT flow diagram.

Fifteen patients participated in this study (Table 1, Figure 1). 

**Figure 2:**
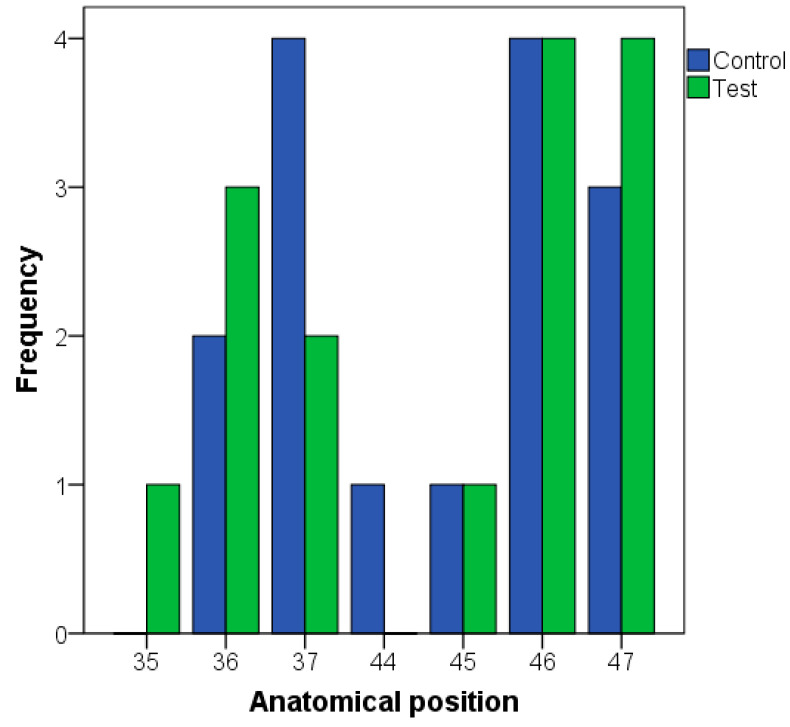
Anatomical position of the dental implants according to the study group.

**Figure 3:**
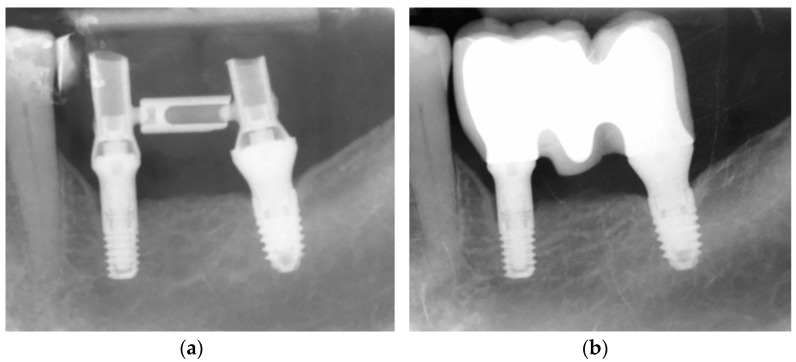
(**a**) Implant placement and immediate loading. #3.6 position implant: 3.0 mm diameter; 5.5 mm length; intermediate abutment height of 4.0 mm. #3.7 position implant: 3.75 mm diameter; 6.5 mm length; intermediate abutment height of 3.5 mm. (**b**) Final restoration one year after implant placement. (Periapical rx).

All the implants were placed in the posterior molar region and there was no statistically significant difference in the anatomical position between the study groups (Table 1 and Figure 2). The implants’ diameters ranged between 3.00 and 4.25 mm. The most common bone type was type I (67% of the implants). Bone density as well as implant insertion torque at the implant position was similar between both groups. The immediate loading protocol was performed in 87% of the implants (Figure 3). The prosthetic (intermediate) abutment length was ≥2 mm in all the implants, being ≥3 mm in 27/30 implants. There were no statistically significant differences between the groups for any variable (Table 2).

Table 3 shows data on the performance of the dental implants. The measurements of the marginal bone level at loading and at 12 months were similar between the study groups without scoring a statistically significant difference. The changes in the marginal bone level indicated limited marginal bone loss in both groups. No implant failure or technical complication was recorded. The peri-implant PPD was <4 mm in both groups and two implants in each group had bleeding on probing. 

## 4. Discussion

Short dental implants offer a promising alternative, reducing surgical complexity and achieving similar or better success rates, compared to longer implants [4,5,6]. Despite variations in defining short-implant length [14,18,19,20], recent studies demonstrate their efficacy and predictability. This study focused on the clinical behavior of 5.5 mm extra-short implants, comparing them to longer implants in the same patients, aiming to further validate their performance. The primary hypothesis was confirmed, as implant length did not show an influence on marginal bone loss. The secondary hypotheses were also confirmed, as implant length did not statistically affect implant survival, peri-implant clinical variables (BoP and PD), or the occurrence of prosthetic complications.

The results of the present study align with those from several SRs [13,14,22,23,24] comparing < 6 mm short implants with longer implants across diverse clinical scenarios, consistently demonstrating better survival rates, marginal bone stability, and fewer mechanical complications for the short-implant groups. Despite the large number of SRs demonstrating the better clinical performance of short implants, another recent SR [30] analyzed only RCTs, comparing short (≤6 mm) and longer implants (>6 mm), and observed contrasting results, leading to some controversy. In that study, longer implant analysis showed significantly higher survival rates (95% CI: 2–5%, *p* < 0.001) in pristine or augmented bone, while MBL and peri-implantitis rates were higher (MBL: 95%CI: −0.17–0.04, *p* > 0.05; peri-implantitis: 95%CI: 0–5%, *p* > 0.05) but not significant for longer implants. No significant differences in technical complications were observed (implant-level 95%CI: −4–6%, *p* > 0.05). Study limitations including variability in sample sizes, patient profiles, bone types, loading protocols, and definitions of peri-implantitis may be related to the discrepancies between different SRs comparing short and longer implants.

The effect of splinting on implant biomechanics should be considered when analyzing the results of this study. Implant-supported single crowns and FPDs differ in biomechanical demands. Single crowns concentrate forces on one implant, increasing stress on that implant and surrounding bone. On the contrary, splinted multiple restorations distribute forces across multiple implants, reducing the load peaks on each peri-implant bone. This distribution mitigates implant overload and contributes to the prevention of MBL. Nevertheless, single crowns on short implants have also shown a good clinical performance even in the more biomechanically demanding scenario of the posterior region [31]. A recent cohort study [32] aimed to compare the survival, MBL, and prosthetic complication rates between short (≤6.5 mm) and longer (≥7.5 mm) implants supporting single-crown restorations in the maxillary/mandibular premolar or molar regions. The study examined 88 short implants (SIs) in 78 patients and 88 long implants (LIs) in 88 patients. All the implants were placed by the same surgeon and restored with the same type of screw-retained restorations on intermediate (transepithelial) abutments. After a median follow-up of 31 months for the SIs and 35 months for the LIs, all implants remained functional, and no significant differences were observed in technical complications (*p* = 0.342) or marginal MBL (*p* = 0.312). Thus, within the retrospective design study limitations, SIs showed similar survival and clinical outcomes to LIs in single-crown restorations in the posterior maxilla/mandible, even in highly demanding clinical scenarios.

The good clinical performance in terms of survival and bone stability for both groups could be partially related to the adherence to minimally invasive approaches, including the low-speed, bone quality-adapted drilling protocol [33]. This drilling protocol adjusts to socket under-preparation to ensure adequate primary stability for successful implant osseointegration and may minimize the damage to the host tissues [27,33]. Long-term studies (>15 years) following the clinical performance of implants placed using this protocol have shown excellent survival and marginal bone stability [34].

The use of screw-retained temporary and definitive prostheses on long intermediate abutments (all ≥2 mm length) could also have contributed to maintaining peri-implant bone stability. A recent 2-year clinical trial [35] highlighted that abutment height is a critical factor influencing MBL around dental implants. Specifically, longer abutments (>2 mm) were associated with significantly lower MBL as shorter abutments reduce supra-crestal tissue height and biological width, providing less protection to the first bone-to-implant contact. Furthermore, a recent SR and meta-analysis [36], aiming to analyze the influence of abutment height on MBL, showed that longer abutments significantly correlated with less MBL (b = −1.630, *p* < 0.003).

The randomized controlled split-mouth trial design of this study allowed for a direct intra-subject comparison between the test and control groups. This approach minimized confounding factors related to biological variability, as each patient serves as their own control. The randomization of implant position further enhanced the reliability of the results by eliminating allocation bias. Another strength was the fact of splinting both test and control implants within the same fixed prostheses (FPDs), ensuring consistent mechanical loading conditions for both groups. This design feature reduced the potential for differential loading, which could otherwise influence the outcomes. The prospective observational component of the study design allowed for the systematic collection and analysis of data over time. Although a one-year observation period is considered a short-term study, it is sufficient to analyze the most critical period for implant integration and initial bone stability after loading. Most complications related to dental implants, such as marginal bone loss, peri-implantitis, and technical complications, typically manifest within the first year. To reduce inter-observer variability, bone stability measurements from radiographic records and clinical assessments were taken by a single, experienced investigator using consistent criteria.

Despite its strengths, this study had some limitations. The inability to blind the surgical team to the implant type due to visible implant length may introduce observer bias. Additionally, reliance on a single investigator for radiographic measurements, while minimizing inter-observer variability, could lead to systematic bias. The focus on posterior regions limits the applicability of the findings to anterior regions, where bone densities and loading conditions differ. Posterior areas were chosen for their higher biomechanical demands, potentially impacting implant performance more than in anterior regions. Lastly, the split-mouth design, while reducing biological variability, may not fully account for systemic factors influencing bone loss and implant survival. Overall, while the study design is robust and offers valuable insights, these limitations should be considered when interpreting the results and their applicability to broader clinical practice.

## 5. Conclusions

The findings of this study suggest that 5.5 mm short implants can clinically perform comparably to longer implants in terms of survival, marginal bone stability, and peri-implant health, providing a viable alternative for certain clinical situations.

## Figures and Tables

**Table 1:** Patient demographics.

Variable	N
Female/male	7/8
Mean age (years; SD)	67 ± 9
Medical History	
Arterial hypertension	5
Hypercholesteremia	4
Allergy to drugs	3
Smoking habit (≤10 cigarettes/day)	2
Extraoral cancer	2
Obstructive sleep apnea	1
Hypothyroidism	1
Diabetes mellites type II	1
Dementia	1
Autoimmune hepatitis	1
Dental History	
Parafunction habits	7
Temporomandibular join disorders	4
Gingivitis	1

**Table 2:** Distribution of implant diameter, anatomical position, bone type, and loading protocol in study groups.

Variable		CONTROL	TEST	*p*-Value
Diameter (mm)	3.00	0	1	0.407 ^a^
	3.30	3	3	
	3.50	5	2	
	3.75	2	5	
	4.00	5	3	
	4.25	0	1	
Anatomical position	Lower premolars	2	2	0.808 ^a^
	Lower molars	13	13	
Bone type	II	10	10	0.574 ^a^
	III	4	5	
	IV	1	0	
Bone density (units) (mean; SD)		636;184	580;110	0.183 ^b^
Insertion torque (Ncm) (median; range)		60; 10 to 70	60; 10 to 65	0.888 ^c^
Loading protocol	Immediate	13	13	0.701 ^d^
	Delayed	2	2	

^a^: Chi square test; ^b^: paired Student test; ^c^: Wilcoxon test; ^d^: Fisher’s exact test; SD: standard deviation.

**Table 3:** Performance data of the dental implants in the study groups. MBL: Marginal bone loss.

Variable		CONTROL	TEST	*p*-Value
Mesial MBL—Loading (mm)	Mean; SD	0.60; 0.62	0.48; 0.39	0.608 ^a^
Mesial MBL—12 months (mm)	Mean; SD	0.40; 0.56	0.26; 0.49	0.430 ^a^
Distal MBL—Loading (mm)	Mean; SD	0.35; 0.48	0.42; 0.48	0.649 ^a^
Distal MBL—12 months (mm)	Mean; SD	0.38; 0.44	0.23; 0.58	0.444 ^a^
Change in mesial MBL (mm)	Median; range	−0.03; −1.75 to 0.45	−0.1; −1.19 to 0.24	0.955 ^b^
Change in distal MBL (mm)	Median; range	0.08; −1.45 to 0.72	−0.05; −1.41 to 0.27	0.118 ^b^
Implant survival		15	15	-
Bleeding on probing—12 months		2	2	-
Pocket proping depth (mm)	Median; range	1.5; 1.0 to 3.0	1.7; 1.3 to 3.2	0.844 ^b^

^a^: Paired Student test; ^b^: Wilcoxon test.

## Data Availability

The datasets used and analyzed during the current study are available from the corresponding author on reasonable request.

## References

[1] Kondo T., Kanayama K., Egusa H., Nishimura I. (2023). Current perspectives of residual ridge resorption: Pathological activation of oral barrier osteoclasts. J. Prosthodont. Res..

[2] Khalifa A.K., Wada M., Ikebe K., Maeda Y. (2016). To what extent residual alveolar ridge can be preserved by implant? A systematic review. Int. J. Implant Dent..

[3] Fanghänel J., Proff P., Dietze S., Bayerlein T., Mack F., Gedrange T. (2006). The morphological and clinical relevance of mandibular and maxillary bone structures for implantation. Folia Morphol..

[4] Terheyden H., Meijer G.J., Raghoebar G.M. (2021). Vertical bone augmentation and regular implants versus short implants in the vertically deficient posterior mandible: A systematic review and meta-analysis of randomized studies. Int. J. Oral Maxillofac. Surg..

[5] Sghaireen M.G., Shrivastava D., Alnusayri M.O., Alahmari A.D., Aldajani A.M., Srivastava K.C., Alam M.K. (2022). Bone Grafts in Dental Implant Management: A Narrative Review. Curr. Pediatr. Rev..

[6] Ravidà A., Serroni M., Borgnakke W.S., Romandini M., Wang I.I., Arena C., Annunziata M., Cecoro G., Saleh M.H.A. (2024). Short (≤6 mm) compared with ≥10-mm dental implants in different clinical scenarios: A systematic review of randomized clinical trials with meta-analysis, trial sequential analysis and quality of evidence grading. J. Clin. Periodontol..

[7] Starch-Jensen T., Nielsen H.B. (2020). Sandwich osteotomy of the atrophic posterior mandible with interpositional autogenous bone block graft compared with bone substitute material: A systematic review and meta-analysis. Br. J. Oral Maxillofac. Surg..

[8] García-Ochoa A.P., Pérez-González F., Moreno A.N., Sánchez-Labrador L., Brinkmann J.C.-B., Martínez-González J.M., Martínez J.L.-Q. (2020). Complications associated with inferior alveolar nerve reposition technique for simultaneous implant-based rehabilitation of atrophic mandibles. A systematic literature review. J. Stomatol. Oral Maxillofac. Surg..

[9] Schwartz S.R. (2020). Short Implants: An Answer to a Challenging Dilemma?. Dent. Clin. N. Am..

[10] Hsu Y.T., Rosen P.S., Choksi K., Shih M.C., Ninneman S., Lee C.T. (2022). Complications of sinus floor elevation procedure and management strategies: A systematic review. Clin. Implant Dent. Relat. Res..

[11] Thoma D.S., Zeltner M., Hüsler J., Hämmerle C.H., Jung R.E. (2015). EAO Supplement Working Group 4—EAO CC 2015 Short implants versus sinus lifting with longer implants to restore the posterior maxilla: A systematic review. Clin. Oral Implant Res..

[12] Kermanshah H., Keshtkar A., Hassani A., Bitaraf T. (2023). Comparing short implants to standard dental implants: A systematic review and meta-analysis of randomized controlled trials with extended follow-up. Evid. Based Dent..

[13] Liang L., Wu X., Yan Q., Shi B. (2024). Are short implants (≤8.5 mm) reliable in the rehabilitation of completely edentulous patients: A systematic review and meta-analysis. J. Prosthet. Dent..

[14] Carosi P., Lorenzi C., Lio F., Laureti M., Ferrigno N., Arcuri C. (2021). Short implants (≤6 mm) as an alternative treatment option to maxillary sinus lift. Int. J. Oral Maxillofac. Surg..

[15] Thoma D.S., Haas R., Sporniak-Tutak K., Garcia A., Taylor T.D., Tutak M., Pohl V., Hämmerle C.H.F. (2024). Randomized controlled multi-centre study comparing shorter dental implants (6 mm) to longer dental implants (11-15 mm) in combination with sinus floor elevation procedures: 10-year data. J Clin Periodontol..

[16] Lai H.C., Si M.S., Zhuang L.F., Shen H., Liu Y.L., Wismeijer D. (2013). Long-term outcomes of short dental implants supporting single crowns in posterior region: A clinical retrospective study of 5–10 years. Clin. Oral Implant Res..

[17] Naenni N., Sahrmann P., Schmidlin P.R., Attin T., Wiedemeier D.B., Sapata V., Hämmerle C.H.F., Jung R.E. (2018). Five-Year Survival of Short Single-Tooth Implants (6 mm): A Randomized Controlled Clinical Trial. J. Dent. Res..

[18] Telleman G., Raghoebar G.M., Vissink A., den Hartog L., Slater J.J.H., Meijer H.J. (2011). A systematic review of the prognosis of short (<10 mm) dental implants placed in the partially edentulous patient. J. Clin. Periodontol..

[19] Wang Y., Jiang J., Guan Y., Si M., He F. (2021). Retrospective Study of Short Versus Standard Posterior Implants and Analysis of Implant Failure Risk Factors. Int. J. Oral Maxillofac. Implant..

[20] Al-Johany S.S., Al Amri M.D., Alsaeed S., Alalola B. (2017). Dental Implant Length and Diameter: A Proposed Classification Scheme. J. Prosthodont..

[21] Hashemi S., Tabatabaei S., Baghaei K., Fathi A., Atash R. (2024). Long-Term Clinical Outcomes of Single Crowns or Short Fixed Partial Dentures Supported by Short (≤6 mm) Dental Implants: A Systematic Review. Eur. J. Dent..

[22] Mendes P.A., Silva V.E.A., da Costa D.V., de Pinho M.M., Chambrone L., Zenóbio E.G. (2023). Effectiveness of Extra-Short (<6 mm) Implants Compared to Standard-Length Implants Associated with Bone Graft: Systematic Review. Int. J. Oral Maxillofac. Implant..

[23] Badaró M.M., Mendoza Marin D.O., Pauletto P., Simek Vega Gonçalves T.M., Porporatti A.L., De Luca Canto G. (2021). Failures in Single Extra-Short Implants (≤6 mm): A Systematic Review and Meta-analysis. Int. J. Oral Maxillofac. Implant..

[24] Grunau O., Terheyden H. (2023). Lateral augmentation of the sinus floor followed by regular implants versus short implants in the vertically deficient posterior maxilla: A systematic review and timewise meta-analysis of randomized studies. Int. J. Oral Maxillofac. Surg..

[25] Guljé F.L., Raghoebar G.M., Gareb B., Vissink A., Meijer H.J.A. (2024). Single crown restorations supported by 6-mm implants in the resorbed posterior mandible: A 10-year prospective case series. Clin Implant Dent Relat Res..

[26] Torgerson D.J., Roberts C. (1999). Understanding controlled trials. Randomisation methods: Concealment. BMJ.

[27] Anitua E., Carda C., Andia I. (2007). A novel drilling procedure and subsequent bone autograft preparation: A technical note. Int. J. Oral Maxillofac. Implant..

[28] Bolle C., Felice P., Barausse C., Pistilli V., Trullenque-Eriksson A., Esposito M. (2018). 4 mm long vs longer implants in augmented bone in posterior atrophic jaws: 1-year post-loading results from a multicentre randomised controlled trial. Eur. J. Oral Implant..

[29] Schulz K.F., Altman D.G., Moher D., CONSORT Group (2010). CONSORT 2010 statement: Updated guidelines for reporting parallel group randomised trials. PLoS Med..

[30] Emfietzoglou R., Dereka X. (2024). Survival Rates of Short Dental Implants (≤6 mm) Used as an Alternative to Longer (>6 mm) Implants for the Rehabilitation of Posterior Partial Edentulism: A Systematic Review of RCTs. Dent. J..

[31] Mangano F.G., Shibli J.A., Sammons R.L., Iaculli F., Piattelli A., Mangano C. (2014). Short (8-mm) locking-taper implants supporting single crowns in posterior region: A prospective clinical study with 1-to 10-years of follow-up. Clin. Oral Implant Res..

[32] Anitua E., Alkhraisat M.H., Eguia A. (2022). Single-crown restorations in premolar-molar regions: Short (≤6.5 mm) vs. longer implants: Retrospective cohort study. Int. J. Implant Dent..

[33] Anitua E., Alkhraisat M.H., Piñas L., Orive G. (2015). Efficacy of biologically guided implant site preparation to obtain adequate primary implant stability. Ann. Anat. Anat. Anz..

[34] Anitua E., Alkhraisat M.H. (2019). Fifteen-Year Follow-up of Short Dental Implants in the Completely Edentulous Jaw: Submerged Versus Nonsubmerged Healing. Implant Dent..

[35] Quispe-López N., Guadilla Y., Gómez-Polo C., López-Valverde N., Flores-Fraile J., Montero J. (2024). The influence of implant depth, abutment height and mucosal phenotype on peri-implant bone levels: A 2-year clinical trial. J. Dent..

[36] Del Amo F.S., Romero-Bustillos M., Catena A., Galindo-Moreno P., Sánchez-Suárez J.M., Sánchez R., Garaicoa-Pazmino C. (2024). Effect of Abutment Height on Marginal Bone Loss Around Dental Implants: A Systematic Review. Int. J. Prosthodont..

